# Redefining the Septal L-Strut in Septal Surgery

**DOI:** 10.1371/journal.pone.0119996

**Published:** 2015-03-24

**Authors:** Jung-Seob Lee, Dong Chang Lee, Dong Heon Ha, Sung Won Kim, Dong-Woo Cho

**Affiliations:** 1 Department of Mechanical Engineering, Postech, Pohang, Korea; 2 Department of Otolaryngology–Head and Neck Surgery, The Catholic University of Korea, College of Medicine, Seoul, Korea; Beijing Institiute of Otolaryngology, CHINA

## Abstract

In septal surgery, the surgeon preserves the L-strut, the portion anterior to a vertical line drawn from the rhinion to the anterior nasal spine (ANS) and at least a 1-cm width of the dorsal and caudal septal segment, to decrease the potential for loss of the tip and dorsal nasal support. However, nasal tip collapse and saddle deformities occur occasionally. We utilized a mechanical approach to determine the safe width size for the L-strut in contact with the maxillary crest. Five L-strut models were designed based on computed tomography data (80 patients) and previous studies (55 patients). All L-strut models connected the perpendicular plate of the ethmoid bone (PPE) and the maxillary crest and were assumed to be fixed to the PPE and maxillary crest. An approximated daily load was applied to the dorsal portion of the L-strut. Finite element analyses were performed to compare the stress, strain, and displacement distribution of all L-strut models. According to the differences in the contact area between the caudal L-strut and maxillary crest, there are significant differences in terms of the stress, strain, and displacement distribution in the L-strut. High stresses occurred at the inner corner of the L-strut when 60 - 100% of the strut was in contact with the maxillary crest. High stresses also occurred at the inferior portion of the caudal L-strut when 20 - 40% of the caudal strut was in contact with maxillary crest. We conclude that it is important to preserve the 1-cm width L-strut caudal segment, which corresponds to the portion posterior to a vertical line drawn from the rhinion to the ANS. In particular, we must maintain more than 40% of the contact area between the L-strut and the maxillary crest when the septal cartilage in the caudal portion of the L-strut is harvested.

## Introduction

Septal surgery is one of the most common procedures performed by rhinologists. In septal surgery, to decrease the potential for loss of the tip and dorsal nasal support, the surgeon preserves at least a 1-cm width portion of the dorsal and caudal septal segment. This portion has been termed the L-strut[1]. Deformities of the septal L-strut create functional and aesthetic problems such as a twisted nose, malpositioned tip, saddle deformity, and internal valve insufficiency[2]. It has been demonstrated that the septal cartilage can be harvested posterior to an imaginary vertical line that is drawn from the rhinion to the anterior nasal spine (ANS) without compromising bridge support for septoplasty or rhinoplasty[3]. However, nasal tip collapse or saddle deformity has been observed occasionally when the septal cartilage was harvested in the caudal portion of the L-strut, which contacts the maxillary crest posterior to the vertical line connecting the rhinion to the ANS.

Few reports have addressed L-strut deformity or collapse from a mechanical perspective[4,5]. There are no published reports on the caudal segment of the septal L-strut posterior to the ANS using this approach[6–8]. Therefore, the purpose of the current study was to identify the pattern of stress distribution of the septal L-strut using finite element method (FEM) analysis. We focused on the inferior portion at which the caudal component of the septal L-strut contacts the maxillary crest. We also redefine the appropriate nomenclature for the L-strut, a structure that must be preserved during septoplasty or rhinoplasty.

## Materials and Methods

### The Finite Element Method

The FEM is a numerical calculation technique to find approximate solutions for boundary value problems using differential equations. It enables the representation of various stress, strain, and energy distributions on a continuum. In this study, a static analysis module from the Ansys Workbench 2.0 Framework (applied Taesung, version 13.0, Korea) was used to obtain the stress, strain, and energy distributions.

### Design of the L-strut model for FEM analysis

The regional angle in the septal L-strut was measured using the computed tomography (CT) findings from patients who underwent transsphenoidal skull base tumor operations at the Seoul St. Mary’s Hospital, Seoul, Korea between July 2008 and September 2011. Approval was obtained from the Institutional Review Board of Catholic university of Korea (KC08FZZZ0302; Analysis of the nasal septum measurement using paranasal sinus computed tomography in the nasal septal deviated patients), and all patients gave written informed consent. A total of 80 patients (male: 39, female: 41) who underwent paranasal sinus computed tomography (PNS CT) imaging for preoperative evaluation were enrolled for examination of the L-strut angle. Exclusion criteria were as follows: previous nasal or sinus surgery, nasal septal deviation, under 20 years of age, anatomic nasal or paranasal variations, or nasal obstructive symptoms. PNS CT images were acquired at 120 kV and 180 mA with a 7-s scanning time. Serial 0.6-mm axial images were obtained, and 1-mm coronal and sagittal slices were reconstructed.

**Fig 1:**
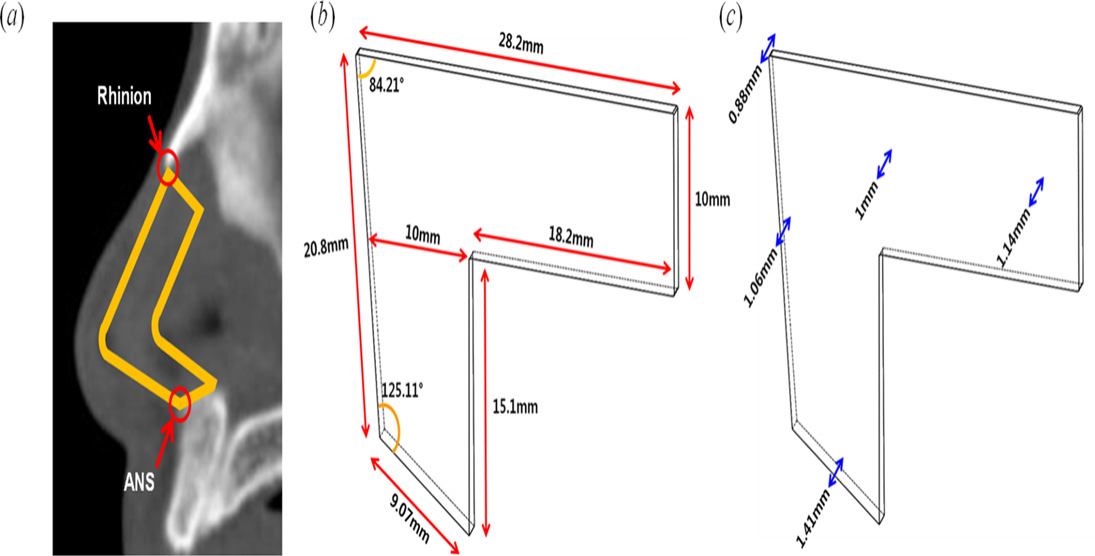
Design of the L-strut model. (*a*) Measurement of angle and specification of the L-strut structure on sagittal nasal septum CT. (*b*) Length and angle of the L-strut model. (*c*) Thickness for design of the L-strut model.

To design the L-strut model, an irregular quadrangle was drawn of which the dorsal and caudal segment composed the two sides. The regional angle data of the septal L-strut were measured in the PNS CT sagittal view that included the nasal septum using the Picture Archiving and Communicating System (PACS) (Marotech, Seoul, Korea). The angle between the dorsal and caudal component of the L-strut and another angle between the caudal segment and maxillary crest were calculated in 80 patients as shown in Fig. 1(*a*). The mean of the two angles was 84.21° and 125.11°, respectively, and these were used to design the L-strut (Fig. 1(*b*)). In addition, the lengths of the dorsal and caudal segments were determined from an intraoperative assessment of 55 Korean patients in a previous report (Fig. 1(*b*))[9]. The L-strut was simultaneously designed to have a 1-cm width (Fig. 1(*a*)). The L-strut thickness was determined from a real cadaver examination value (Fig. 1(*c*))[10].

### Material properties of the septal cartilage in FEM analysis

The compressive modulus and Poisson’s ratio were set to 0.41 MPa and 0.3, respectively (Table 1). The compressive modulus and Poisson’s ratio were chosen from studies in which the mechanical properties of the septal cartilage were reported[11,12]. The compressive modulus and Poisson’s ratio of the septal cartilage have a range of 0.4 MPa to 19.3 kPa and 0.26 to 0.44, respectively[5,11–13].

**Table 1:** Engineering data.

Young’s modulus	4.1 x 10^5^ (Pa)
Poisson’s ratio	0.3

### Boundary conditions in FEM analysis

**Fig 2:**
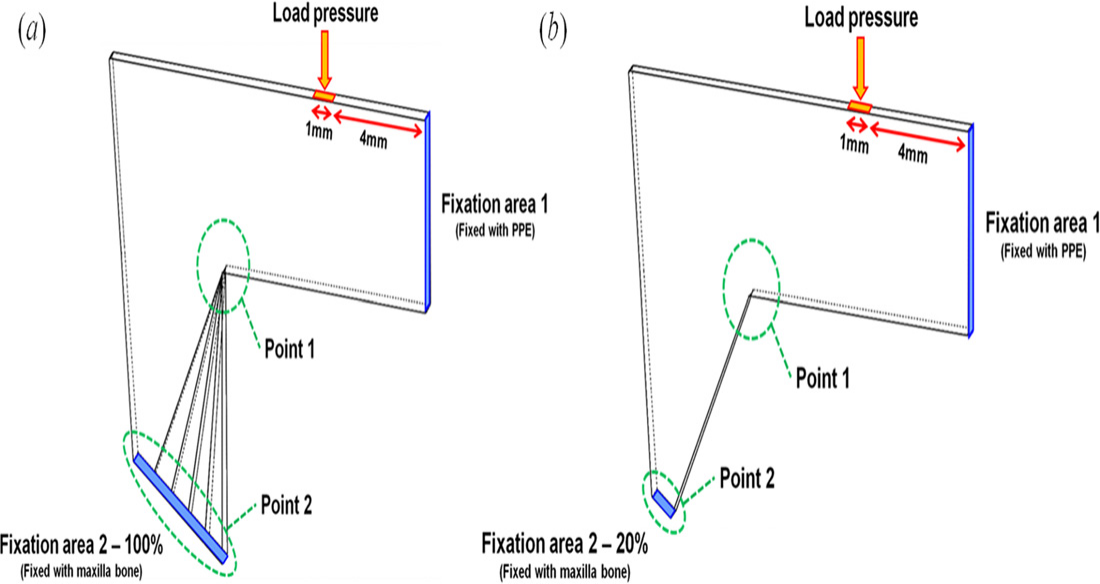
Boundary conditions of the L-strut for stress, strain energy, and displacement analysis. (*a*) 100% FA-2 fixation condition. (*b*) 20% FA-2 fixation condition.

The L-strut environment in the nasal septum was considered to determine the boundary conditions for exact analysis. Two fixation conditions of the L-strut were chosen and termed fixation area 1 (FA-1) and fixation area 2 (FA-2) for convenience of analysis. FA-1 and FA-2 of the L-strut are the contact areas between the perpendicular plate of the ethmoid bone (PPE) and the L-strut, and the maxillary crest and the L-strut, respectively (Fig. 2). FA-1 is considered fully bound together because the keystone area is conserved. FA-2 was classified as 20%, 40%, 60%, 80%, or 100% contact between the strut and maxillary crest.

When the caudal segment of the L-strut in contact with the maxillary bone was completely preserved during the septal surgery, the contact part was termed the 100% FA-2 condition (Fig. 2(*a*)). The 20%, 40%, 60%, and 80% FA-2 conditions were a ratio of the contact area to the standard area of 100%. Therefore, 20%, 40%, 60%, and 80% FA-2 meant that the septal cartilage in contact with the maxillary bone was partly harvested behind the ANS. Fig. 2(*b*) represents 20% fixation at FA-2. According to various FA-2 conditions, static analysis of the L-strut was conducted and the stress, strain, and displacement distribution were calculated.

The loading condition was determined as the load (0.04N) that eyeglasses exert on the nose[14]. The loading position was set at 4 mm along the nasal dorsum from FA-1 and was exerted perpendicularly to the dorsal segment of the L-strut, as shown in Fig. 2. The stress, the strain, and the displacement of the L-strut were analyzed at Point 1 and Point 2 on the L-strut model (Fig. 2). Point 1 and 2 were defined as the inner corner of the L-strut and the point of contact with the maxillary crest, respectively, because high stress and displacement values occur at these locations.

## Results

### Stress distribution of L-strut

**Fig 3:**
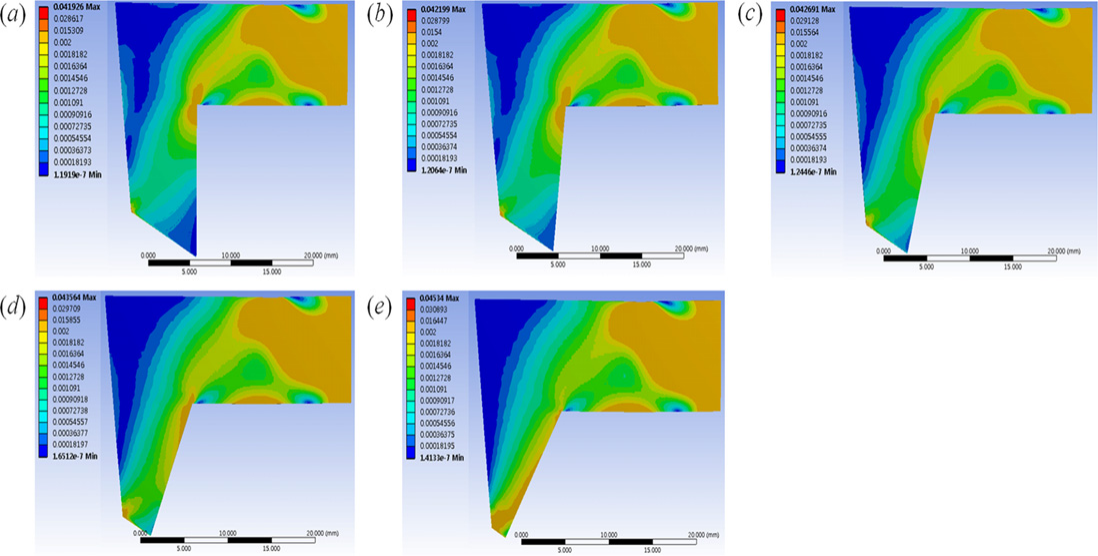
Stress distribution results of L-strut. The figure represent the stress distribution in the (*a*)100%, (*b*)80%, (*c*)60%, (*d*)40%, and (*e*)20% FA-2 conditions(contact area between the caudal segment of the L-strut and maxillary crest) under the same loading condition (the load (0.04N) that eyeglasses exert on the nose).

Fig. 3 shows the stress distribution of the L-strut with various FA-2 conditions under the same loading condition (0.04 N). Shades of blue and red correspond to lower and higher stresses, respectively. When the septal cartilage segment was harvested, the decreased contact area between the caudal segment of the L-strut and maxillary crest resulted in increased stress on the L-strut. The increased stress occurred from Point 1 to FA-1 with the 60–100% FA-2 conditions. With the 20–40% FA-2 conditions, the increased stress was generated from Point 1 to Point 2, and the maximum stress occurred at Point 2. The maximum stress began to occur at Point 2, and high stress occurred evenly along the caudal segment of the L-strut in the 20–40% FA-2 conditions. When FA-2 had 20% fixation, the stress rapidly increased at Point 2. The stress distribution near FA-1 maintained a constant value regardless of FA-2 conditions.

### Stress, strain energy, and displacement analysis of the L-strut

**Fig 4:**
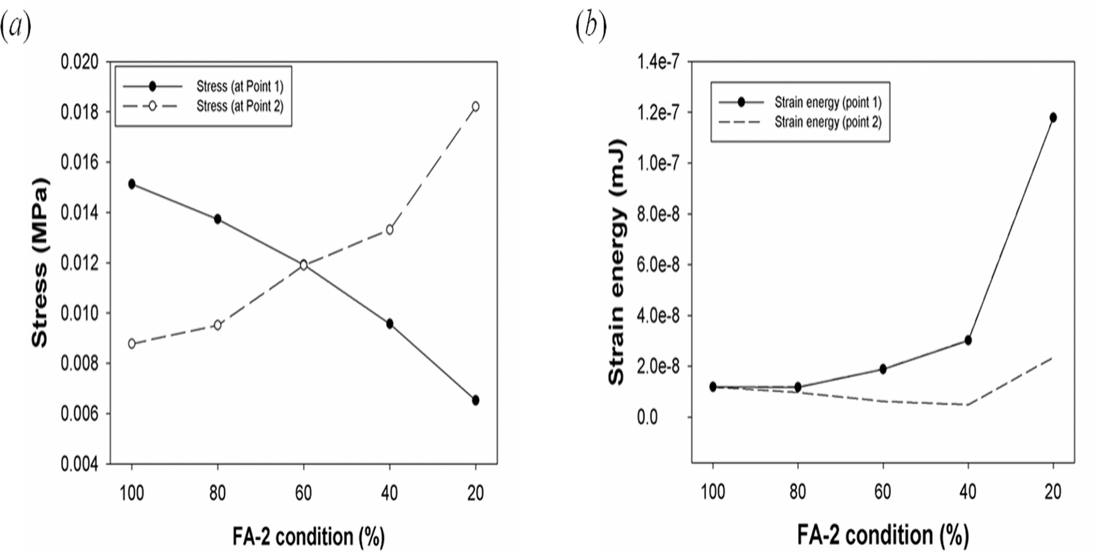
Graphs of stress and strain energy of the L-strut. Graphs of (*a*) stress and (*b*) strain energy of the L-strut according to different FA-2 conditions.

Fig. 4(*a*) shows the stress values according to FA-2 conditions. The maximum stresses at Points 1 and 2 were 0.0151 and 0.0087735 MPa in the 100% FA-2 condition, respectively. The maximum stress values were identical in the 60% FA-2 condition, as shown in Fig. 4(*a*). The maximum stress in the 80–100% FA-2 conditions and 20–40% FA-2 conditions occurred at Point 1 and Point 2, respectively. Fig. 4(*b*) shows the maximum strain energies at Point 1 and Point 2, along with the FA-2 conditions. The maximum strain energies at Point 1 and Point 2 occurred in the 20% FA-2 condition. There was similar strain energy in the 60–100% FA-2 conditions, and the strain energy in the 20–40% FA-2 conditions increased rapidly. A large deformation of the L-strut occurred when there was a less than 40% contact area between the L-strut and maxillary bone.

**Fig 5:**
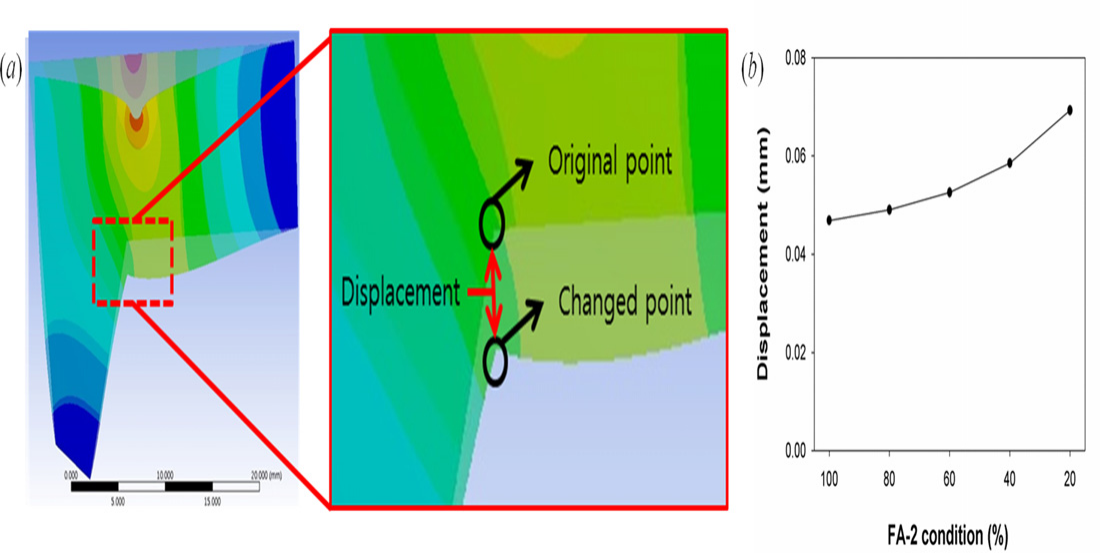
The figure of displacement of L-strut and the maximum values of displacement. (*a*) Explanation of displacement of L-strut. (*b*) Graph of the maximum values of displacement at point 1 (the inner angle area of the L-strut) according to the FA-2 condition.

Fig. 5 shows the L-strut displacement at Point 1. The displacement was measured before and after loading the L-strut; this comparison is shown in Fig. 5*(a)*. The L-strut displacement values according to FA-2 conditions at Point 1 are shown in Fig. 5(*b*). As the contact area between the L-strut and maxillary bone decreased, the displacement values increased.

## Discussion

Excessive loads and unrealistic L-strut designs were used in some previous reports[4,5]. However, in this study, we assumed that the force glasses exert on the septum from the bridge of the nose could be considered an ordinary force. Moreover, the thickness of the septum differs from that in previous models and is closer to the real anatomical dimensions. The angle of the L-strut was also measured using the mean value calculated using PNS CT from 80 patients. The length of the septum was determined using the average value from intraoperative measurements in 55 patients[9]. Thus, these parameters increase in the reliability of the FEM. Therefore, the FEM results may predict postoperative septal deformities from common loading forces or augmentation materials after the septal harvest in septoplasty or rhinoplasty.

When we carried out FEM analysis with the loading forces applied to the dorsal segment of the L-strut, the maximum stress appeared on the inner angle (Point 1) of the L-strut if the caudal strut was intact. If the contact area between the L-strut and the maxillary bone decreased to below 40%, the stress on the L-strut greatly increased at the caudal segment in contact with maxillary crest (Point 2). At Point 2, the stress values produced in the 60–100% FA-2 conditions were less than those produced in the 20–40% FA-2 conditions, and maximum stress occurred in the 20–40% FA-2 conditions.

The strain energy at the caudal segment of the L-strut in contact with the maxillary bone greatly increased in the 20–40% FA-2 conditions. However, the strain energy was relatively low in the 60–100% FA-2 conditions. We found that the smaller contact area is, the larger the displacement at Point 1 of the L-strut. Therefore, the possibility of L-strut collapse was the greatest in the 20% FA-2 condition. In the 40% FA-2 condition, there was also the possibility of L-strut collapse considering the stress distribution, strain energy, and displacement. In the 20–40% FA-2 conditions, the caudal segment of the L-strut connected to the maxillary bone had relatively high stress and strain energy. In the 60–100% FA-2 conditions, the dorsal segment of the L-strut connected to the PPE had relatively high stress; however, the dorsal and caudal segments of the L-strut had low strain energy. Therefore, the possibility of collapse and deformity of the shape of the L-strut might be higher in the 20–40% FA-2 conditions than in the 60–100% FA-2 conditions[15–16].

According to the results of this study, it is important to preserve the 1-cm width of the inferior portion of the caudal L-strut segment, which is also the portion posterior to a vertical line drawn from the rhinion to the ANS. The risk of developing a saddle deformity and nasal tip ptosis increases when the portion in contact with the inferior part of caudal segment and the maxillary crest is below 40% when the L-strut width is 1 cm. Thus, when we harvest the septal cartilage from the caudal portion of the L-strut, we must preserve the width of caudal segment so that more than 40% of the caudal strut is in contact with the maxillary crest. Moreover, we should not excessively resect this interface when correcting a caudal septal deviation for septal straightening. Inevitably, if we should excessively removed this interface in anterior septoplasty with swing door technique, subtotal or total extracorporeal septoplasty for severe deviation, we must refixate more than 40% of the caudal strut at the maxillary crest for reestablishment.

This study was conducted to analyze the septal L-strut without considering any other factors that could influence strut support. Dorsally and caudally, the cartilaginous septum is interconnected with the upper lateral cartilages and lower lateral cartilages, respectively[2,17]. Thus, the upper and lower lateral cartilages may also be important for septal cartilage mechanical support. Moreover, the membranous septal cartilage composes the support mechanism for septum. Septal lining flaps serve as soft tissue braces across the intrinsically weak interface at the bone-cartilage junction. The perichondrial layer of the septal lining flap has been shown to account for the majority of the flap’s biomechanical strength[18]. The caudal edge of the bony vault in the midline extends past the bone-cartilage junction caudally in most individuals. The overlap with the dorsal strut and the bony vault also contributes to L-strut stability[4]. In addition, a weak fixation of the L-strut to the ANS and the cephalic rotation of the L-strut would influence the loss of tip support and the loss of dorsal septal height.

To model more realistically the mechanical behavior of the L-strut using FEM, additional studies incorporating boundary conditions from the surrounding environment in the nasal septum are needed. The nasal septum is also supported by the upper lateral cartilage, the lower lateral cartilage, the membranous septal cartilage, the septal flap, and the overlap with the bony vault. Therefore, those structures influence L-strut support. Various loading forces, evaluation points, and directional forces should be used to yield more realistic results. The precise measurement of many parameters (e.g., material properties) that can affect the nose and the septal cartilage is required for future FEM analysis.

## Conclusion

We found that the inferior portion of the L-strut must be preserved when septal cartilage harvest is performed. Thus, the septal L-strut must be redefined as a 1-cm width from the ANS, and not a 1-cm width posterior from the caudal end in the septal cartilage, as previously described. When septoplasty or rhinoplasty is performed, the caudal segment of the L-strut in contact with the maxillary crest posterior to an imaginary vertical line connecting the rhinion to the ANS must be conserved. When the inferior portion of caudal strut is resected, more than 40% of the L-strut width must be conserved to avoid nasal tip ptosis or a saddle deformity. In the future, further studies should utilize FEM to evaluate the mechanical properties of nasal structures because computer simulations are powerful tools with which to analyze septal mechanics[19].
