# Correction: Redefining the Septal L-Strut in Septal Surgery

**DOI:** 10.1371/journal.pone.0128284

**Published:** 2015-05-08

**Authors:** Jung-Seob Lee, Dong Chang Lee, Dong-Heon Ha, Sung Won Kim, Dong-Woo Cho

The third author’s name is incorrect. The correct name is: Dong-Heon Ha. The correct citation is: Lee J-S, Lee DC, Ha D-H, Kim SW, Cho D-W (2015) Redefining the Septal L-Strut in Septal Surgery. PLoS ONE 10(3): e0119996. doi:10.1371/journal.pone.0119996
